# Unbounded solutions of models for glycolysis

**DOI:** 10.1007/s00285-021-01560-y

**Published:** 2021-01-19

**Authors:** Pia Brechmann, Alan D. Rendall

**Affiliations:** Institut für Mathematik Johannes Gutenberg-Universität, Staudingerweg 9, 55099 Mainz, Germany

**Keywords:** Glycolysis, Oscillations, Dynamical system, 92C40, 34C11, 37N25

## Abstract

The Selkov oscillator, a simple description of glycolysis, is a system of two ordinary differential equations with mass action kinetics. In previous work the authors established several properties of the solutions of this system. In the present paper we extend this to prove that this system has solutions which diverge to infinity in an oscillatory manner at late times. This is done with the help of a Poincaré compactification of the system and a shooting argument. This system was originally derived from another system with Michaelis–Menten kinetics. A Poincaré compactification of the latter system is carried out and this is used to show that the Michaelis–Menten system, like that with mass action, has solutions which diverge to infinity in a monotone manner. It is also shown to admit subcritical Hopf bifurcations and thus unstable periodic solutions. We discuss to what extent the unbounded solutions cast doubt on the biological relevance of the Selkov oscillator and compare it with other models for the same biological system in the literature.

## Introduction

When trying to understand a biological system with the help of mathematical modelling it often happens that there are several different models for the same biological situation in the literature. In view of this it is important to have criteria for deciding between models. One strategy for identifying criteria of this type is to look at relatively simple examples in great detail. In order to do this effectively it is necessary to have a sufficiently comprehensive understanding of the properties of solutions of the models being studied. In this paper, with this strategy in mind, we look in detail at the dynamical properties of certain models for glycolysis.

Glycolysis is part of the process by which living organisms extract energy from sugar (Alberts et al. 2008). A suitable model system for studying this phenomenon experimentally is yeast extract or a suspension of yeast cells. The first indication that this system might have interesting dynamical properties was given by damped oscillations reported in Duysens and Amesz (1957). Later it was discovered that a constant continuous supply of sugar can lead to sustained oscillations (cf. Boiteux et al. 1975). Looking for the source of these oscillations revealed that they are produced by a small reaction network describing the action of the enzyme phosphofructokinase. A mathematical model for this network was set up and studied by Higgins (1964). It was found by Selkov that this model was not adequate for describing the oscillations and he introduced a modified one (Selkov 1968). The starting point for the model of Selkov is a reaction network with five chemical species. Assuming mass action kinetics leads to a system of five ordinary differential equations. Using quasi-steady state assumptions this can be reduced to a system of two equations with nonlinearities of Michaelis–Menten type. For brevity we call it ’the Michaelis–Menten system’ in what follows. Setting one of the coefficients in this system to zero leads to a further simplification, giving a system of two equations with mass action kinetics, which we call the ’basic Selkov system’ in what follows.

The aim of this paper is to obtain a better understanding of the dynamics of solutions of the three systems just described. A number of properties of solutions of the basic Selkov system were already established in Selkov (1968) but for many years no further rigorous results on this subject were obtained. Important progress was made in a paper of d’Onofrio (2010) and a number of additional properties of the solutions were established in a recent paper of the authors (Brechmann and Rendall 2018). In particular it was proved that for any values of the parameters there exist unbounded solutions of this system which are eventually monotone in the sense that for a solution of this type both concentrations are monotone after a certain time. In Selkov (1968) it is claimed that this system admits solutions which oscillate with an amplitude which grows without limit at late times. In what follows solutions of this type are referred to as ’solutions with unbounded oscillations’. The paper Selkov (1968) provides no justification for the claim other than a mention of numerical simulations, about which no details are given. Up to now there was no proof of the truth or falsity of this claim of Selkov (1968). One of the main results of the present paper is a proof of the existence of solutions of the basic Selkov system with unbounded oscillations. Our discovery of this proof was stimulated by the paper (Merkin et al. 1987), which belongs to the domain of theoretical chemistry. It deals with a system which turns out to be identical to the basic Selkov system when a parameter $\gamma$ in the latter system takes the value two.

In Merkin et al. (1987) a claim of the existence of solutions with unbounded oscillations is also made. It is supported by an intricate heuristic argument using matched asymptotic expansions. It is not at all clear how this argument could be translated into a rigorous one but it provided us with some ideas which, when combined with the results of Brechmann and Rendall (2018), do give a proof of the existence of solutions with unbounded oscillations. When written in dimensionless form the system contains one parameter $\alpha$. As claimed in Merkin et al. (1987), solutions with unbounded oscillations occur for precisely one value $\alpha _1$ of $\alpha$. When $\alpha$ is slightly less than $\alpha _1$ there exists a stable periodic solution. As $\alpha$ approaches $\alpha _1$ from below the amplitude of the periodic solution tends to infinity. One important element of this proof is to study the limit of the system for $\alpha \rightarrow \infty$ after a suitable rescaling. The existence of $\alpha _1$ is then proved by a shooting argument. A monotonicity property, which was apparently not previously known, is used to obtain the uniqueness of $\alpha _1$.

The presence of unbounded solutions, whether monotone or oscillatory, might be seen as a feature which is unrealistic from the point of view of the biological applications. The monotone unbounded solutions of the basic Selkov system are not mentioned at all in Selkov (1968). That system is the limit of the Michaelis–Menten system when a parameter $\nu$ tends to zero. It is stated in Selkov (1968) that solutions with unbounded oscillations do not exist for $\nu >0$. On the other hand simulations reported in Keener and Sneyd (2009) suggest that the amplitude of periodic solutions of the Michaelis–Menten system diverges rapidly to infinity when a parameter is varied in a finite range. This indicates that, in contrast to the claim of Selkov, the existence of unbounded oscillations is a phenomenon which may persist for $\nu >0$. If this is true then the presence of these biologically problematic solutions of the basic Selkov system is not just an artefact of taking the limit $\nu \rightarrow 0$. The issue of the existence of solutions with unbounded oscillations in the case of the Michaelis–Menten system is not resolved in what follows but some partial results are obtained. In particular it is shown that for the Michaelis–Menten system with arbitrary parameters there are unbounded solutions which are eventually monotone and whose leading order asymptotics are identical to those found in the basic Selkov system. It is also shown that for certain combinations of the parameters $(\alpha ,\nu )$ with $\nu >0$ all positive solutions except the steady state have these late-time asymptotics. It turns out that there are parameter values for which there exist unstable periodic solutions of the Michaelis–Menten system. This is in contrast to the basic Selkov system where it was proved in Brechmann and Rendall (2018) that all periodic solutions are asymptotically stable.

The structure of the paper is as follows. The various systems considered in the paper are defined in Sect. 2. In Sect. 3, after some necessary results on the basic Selkov system proved in Brechmann and Rendall (2018) have been recalled, the existence of solutions with unbounded oscillations is proved. Similarities and differences between the properties of solutions of the basic Selkov system and the Michaelis–Menten system are discussed in the next three sections. Section 4 discusses the Hopf bifurcation exhibited by the Michaelis–Menten system. Its Poincaré compactification is computed in Sect. 5. Global properties of the Michaelis–Menten system are discussed in Sect. 6. The paper ends with a conclusion and outlook.

## Survey of the systems considered

In Selkov (1968) a simple reaction network describing glycolysis is introduced. Assuming mass action kinetics for this network leads to a system of five ordinary differential equations, system (4) of Selkov (1968). In a slightly modified notation this system is

$$\begin{aligned} \frac{ds_1}{dt}= & {} v_1-k_1s_1x_1+k_{-1}x_2,\\ \frac{ds_2}{dt}= & {} k_2x_2-\gamma k_3s_2^\gamma e+\gamma k_{-3}x_1-v_2s_2,\\ \frac{dx_1}{dt}= & {} -k_1s_1x_1+(k_{-1}+k_2)x_2+k_3s_2^\gamma e-k_{-3}x_1,\\ \frac{dx_2}{dt}= & {} k_1s_1x_1-(k_{-1}+k_2)x_2,\\ \frac{de}{dt}= & {} -k_3s_2^\gamma e+k_{-3}x_1. \end{aligned}$$

In fact a factor $\gamma$ was omitted in two places in Selkov (1968) and this error has been corrected here. All the parameters are positive and it is assumed that $\gamma >1$, which encodes the biological property of cooperativity. Note that $e_0=e+x_1+x_2$ is a conserved quantity (total amount of enzyme) and this can be used to eliminate *e* from the first four evolution equations and discard the evolution equation for *e*. This reduces the system to four equations.

Dimensionless variables can be introduced by defining

$$\begin{aligned} \sigma _1=\frac{k_1s_1}{k_{-1}+k_2},\quad \sigma _2=\left( \frac{k_3}{k_{-3}}\right) ^{\frac{1}{\gamma }}s_2,\quad u_1=\frac{x_1}{e_0},\ u_2=\frac{x_2}{e_0},\quad \theta =\frac{e_0k_1k_2}{k_{-1}+k_2}t. \end{aligned}$$

This leads to the system

1 $$\begin{aligned} \frac{d\sigma _1}{d\theta }= & {} \nu -\frac{k_2+k_{-1}}{k_2}u_1\sigma _1 +\frac{k_{-1}}{k_2}u_2, \end{aligned}$$

2 $$\begin{aligned} \frac{d\sigma _2}{d\theta }= & {} \eta \left( u_2-\gamma \frac{k_{-3}}{k_2} \sigma _2^\gamma (1-u_1-u_2)+\gamma \frac{k_{-3}}{k_2}u_1-\chi \sigma _2\right) , \end{aligned}$$

3 $$\begin{aligned} \epsilon \frac{du_1}{d\theta }= & {} u_2-\sigma _1u_1+\frac{k_{-3}}{k_2+k_{-1}} (\sigma _2^\gamma (1-u_1-u_2)-u_1),\end{aligned}$$

4 $$\begin{aligned} \epsilon \frac{du_2}{d\theta }= & {} \sigma _1u_1-u_2 \end{aligned}$$

where

$$\begin{aligned} \epsilon= & {} \frac{e_0k_1k_2}{(k_2+k_{-1})^2},\quad \nu =\frac{v_1}{k_2e_0}, \eta =\frac{k_2+k_{-1}}{k_1}\left( \frac{k_3}{k_{-3}}\right) ^{\frac{1}{\gamma }},\quad \chi =\frac{v_2}{k_2e_0}\left( \frac{k_{-3}}{k_3}\right) ^{\frac{1}{\gamma }}. \end{aligned}$$

Formally setting $\epsilon =0$ in the Eqs. (3) and (4) gives $u_2=\sigma _1 u_1$ and $u_1=\frac{\sigma _2^\gamma }{1+\sigma _2^\gamma +\sigma _1\sigma _2^\gamma }$ and substituting these relations into the evolution equations for $\sigma _1$ and $\sigma _2$ gives

5 $$\begin{aligned} \frac{d\sigma _1}{d\theta }= & {} \nu -\left( \frac{\sigma _1\sigma _2^\gamma }{1+\sigma _2^\gamma +\sigma _1\sigma _2^\gamma }\right) , \end{aligned}$$

6 $$\begin{aligned} \frac{d\sigma _2}{d\theta }= & {} \eta \left( \frac{\sigma _1\sigma _2^\gamma }{1+\sigma _2^\gamma +\sigma _1\sigma _2^\gamma } -\chi \sigma _2\right) . \end{aligned}$$

As has been discussed in Brechmann and Rendall (2018) geometric singular perturbation theory (GSPT) can be used to show that solutions of (1)–(4) converge to solutions of (5) and (6) in the limit $\epsilon \rightarrow 0$.

In Selkov (1968) a further simplification of this system is introduced. Consider the rescaled quantities

$$\begin{aligned} x=\frac{\nu ^{\gamma -1}}{\chi ^\gamma }\sigma _1,\quad y=\frac{\chi }{\nu }\sigma _2,\quad \alpha =\frac{\eta \chi ^{\gamma +1}}{\nu ^\gamma },\quad \beta =\frac{\nu ^{\gamma -1}}{\chi ^\gamma },\quad \tau =\left( \frac{\nu }{\chi }\right) ^\gamma \theta . \end{aligned}$$

Expressing the Eqs. (5) and (6) in terms of these gives

7 $$\begin{aligned} \frac{dx}{d\tau }= & {} 1-\frac{xy^\gamma }{1+\nu y^\gamma (\beta +x)}, \end{aligned}$$

8 $$\begin{aligned} \frac{dy}{d\tau }= & {} \alpha \left[ \frac{xy^\gamma }{1+\nu y^\gamma (\beta +x)}-y\right] . \end{aligned}$$

This system has a regular limit when $\nu$ tends to zero with $\alpha$ and $\beta$ fixed. In the limit we get the basic Selkov system, system (II) of Selkov (1968), which is

9 $$\begin{aligned} \frac{dx}{d\tau }= & {} 1-xy^\gamma , \end{aligned}$$

10 $$\begin{aligned} \frac{dy}{d\tau }= & {} \alpha y(xy^{\gamma -1}-1). \end{aligned}$$

It is the system of central interest in Selkov (1968) and the dynamical properties of its solutions are studied in detail in Brechmann and Rendall (2018). Of course (9) and (10) can be thought of as the special case of (7) and (8) where $\nu =0$. Note that it follows from (7) and (8) that $\frac{d}{d\tau }(\alpha x+y)=\alpha (1-y)$. Since any solution with positive initial data remains positive as long as it exists this relation shows that it remains bounded on any finite time interval. Hence, when maximally extended, it exists globally in the future.

## The basic Selkov system

The following proposition collects some of the properties of solutions of the basic Selkov system established in Brechmann and Rendall (2018).

### Proposition 1

The basic Selkov system (9) and (10) has the following properties.

1. For each value of the parameter $\alpha$ the unique positive steady state $P_0$ has coordinates (1, 1).
2. For each $\alpha \in \left( 0,\frac{1}{\gamma -1}\right)$ the positive steady state $P_0$ is asymptotically stable and there exist no periodic solutions.
3. For $\alpha =\alpha _0=\frac{1}{\gamma -1}$ a generic supercritical Hopf bifurcation occurs.
4. For each value of the parameter $\alpha$ there exist positive numbers $x_0$ and $y_0$ such that if a solution satisfies $x(t)\ge x_0$ and $y(t)\le y_0$ at some time *t* it satisfies $\dot{x}(t)>0$ and $\dot{y}(t)<0$ at all later times, $\lim _{t\rightarrow \infty }x(t)=\infty$ and $\lim _{t\rightarrow \infty }y(t)=0$.

**Fig. 1:**
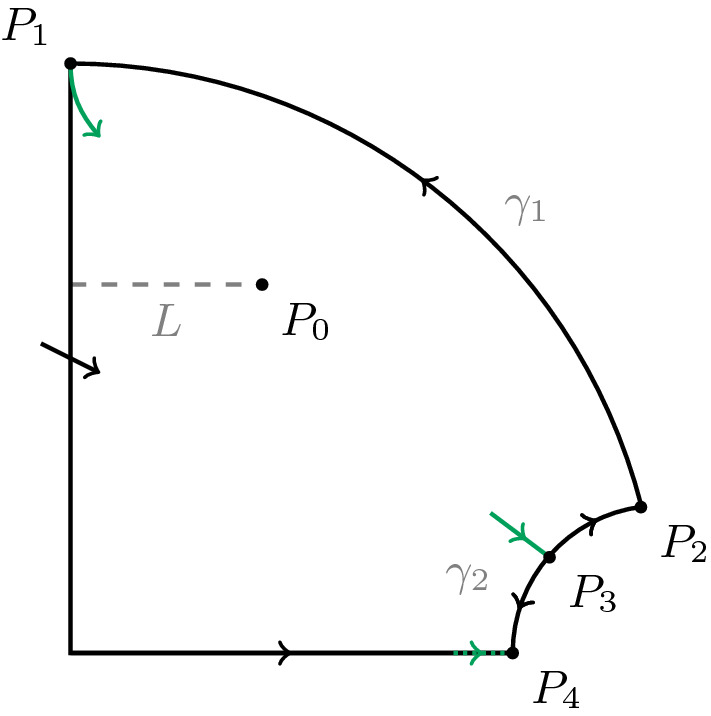
Poincaré compactification of the basic Selkov system (9) and (10)

Using the standard theory of Hopf bifurcations it follows from statement 3. of the proposition that for any $\alpha$ slightly greater than $\alpha _0$ there exists a stable periodic solution. For the convenience of the reader we recall one of the main results of Brechmann and Rendall (2018) (Theorem 3 of that paper). The points $P_i$ in the statement are those occurring in Fig. 1 and whose definition is explained below.

### Theorem 1

In the case $\alpha >\alpha _0$ of the system (9) and (10) exactly one of the following three situations occurs

1. The centre manifolds of $P_1$ and $P_3$ coincide so that there is a heteroclinic cycle at infinity. Any solution which starts below this centre manifold converges to $P_4$ as $t\rightarrow \infty$ while any solution other than the steady state which starts above this manifold converges to the heteroclinic cycle at infinity as $t\rightarrow \infty$.
2. The centre manifolds of $P_1$ and $P_3$ do not coincide. There exists a unique periodic solution. Any solution which starts below the centre manifold of $P_3$ converges to $P_4$ as $t\rightarrow \infty$ while any solution other than the steady state which starts above this manifold converges to the periodic solution as $t\rightarrow \infty$.
3. The centre manifolds of $P_1$ and $P_3$ do not coincide. Any solution other than the steady state which does not lie on the centre manifold of $P_3$ converges to $P_4$ as $t\rightarrow \infty$.

A key question left open in Brechmann and Rendall (2018) is that of what happens to the periodic solution when $\alpha$ gets large. This question is answered in this section.

### Theorem 2

There exists a number $\alpha _1>\alpha _0$ such that the basic Selkov system (9) and (10) has the following properties.

1. For $\alpha =\alpha _1$ there exist solutions with the properties that $\liminf _{t\rightarrow \infty }x(t)=\liminf _{t\rightarrow \infty }y(t)=0$ and $\limsup _{t\rightarrow \infty }x(t)=\limsup _{t\rightarrow \infty }y(t)=\infty$.
2. For $\alpha _0<\alpha <\alpha _1$ there exists a unique periodic solution and it is asymptotically stable.
3. For $\alpha >\alpha _1$ each solution other than the steady state $P_0$ is unbounded and has the properties described in statement 4. of Proposition 1.
4. As $\alpha$ tends to $\alpha _1$ from below the diameter of the image of the periodic solution tends to infinity.

**Fig. 2:**
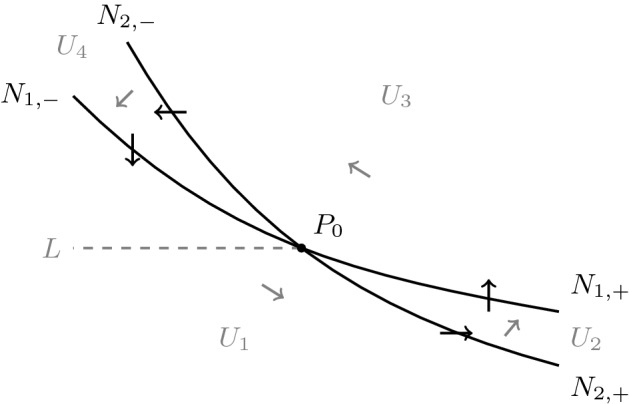
Nullclines of the basic Selkov system (9) and (10)

We adopt some of the notation of Brechmann and Rendall (2018). There the Poincaré compactification of the basic Selkov system is computed and one of the resulting points at infinity is blown up. After this has been done there are four steady states at infinity called $P_1$, $P_2$, $P_3$ and $P_4$. Their positions can be seen in Fig. 1, which is a modification of Fig. 1 of Brechmann and Rendall (2018). Each of the points $P_1$ and $P_3$ has a one-dimensional centre manifold with the flow on the centre manifolds being away from $P_1$ and towards $P_3$. As a starting point for the proof of Theorem 2 we establish some further properties of the centre manifolds of the points $P_1$ and $P_3$, both of which are unique. Let *L* be the segment of the line $y=1$ where $0<x\le 1$. We use the notation for the components $U_i$ of the complement of the nullclines $N_1=N_1^+\cup N_1^-$ and $N_2=N_2^+\cup N_2^-$ which can be seen in Fig. 2, a modification of Fig. 2 of Brechmann and Rendall (2018). Here $N_1$ and $N_2$ are the zero sets of $\frac{dx}{d\tau }$ and $\frac{dy}{d\tau }$, respectively.

### Lemma 1

In the basic Selkov system the centre manifolds of $P_1$ and $P_3$ both contain a point of *L* in their closures.

### Proof

For a point on the centre manifold of $P_1$ sufficiently near to $P_1$ we have $\dot{x}>0$. Hence the manifold initially lies in the region $U_1$. As long as $x<1$ it must remain in $U_1$ and both coordinates of a solution on the centre manifold are monotone functions of time. Hence a solution on the centre manifold of $P_1$ either reaches a point of *L* with $x<1$ after a finite time or it tends to the positive steady state as $t\rightarrow \infty$. Similarly a solution on the centre manifold of $P_3$, when followed backwards in time, either reaches a point of *L* with $x<1$ after a finite time or it tends to the positive steady state as $t\rightarrow -\infty$. $\square$

For a given value of $\alpha$ let $\xi _1(\alpha )$ be the *x*-coordinate of the point where the centre manifold of $P_1$ meets *L* if such a point exists and otherwise let $\xi _1(\alpha )=1$. Define $\xi _2(\alpha )$ similarly in terms of the centre manifold of $P_3$. Note that each centre manifold depends smoothly on the parameter $\alpha$, in the sense that we can choose initial data for solutions on the centre manifold for different values of $\alpha$ in such a way that the solutions depend smoothly on $\alpha$. This can be seen by considering the suspended system obtained by adjoining the equation ${\dot{\alpha }}=0$ to the basic Selkov system and noting that it has a two-dimensional centre manifold at the points corresponding to $P_1$ and $P_3$. This manifold is foliated by curves of constant $\alpha$ which are centre manifolds for the original system. Their smooth dependence on $\alpha$ follows from the smoothness of the two-dimensional centre manifold.

### Lemma 2

The function $\xi _1-\xi _2$ describing the separation of the points where the centre manifolds of $P_1$ and $P_3$ reach $y=1$ is continuous.

### Proof

Consider a value of $\alpha _c$ for which $\xi _1(\alpha _c)<1$. The centre manifold for that value crosses *L* transversely and so, by the implicit function theorem, $\xi _1$ is a smooth function of $\alpha$ close to $\alpha _c$. This also shows that the set of values of $\alpha$ for which $\xi _1(\alpha )<1$ is open. Consider now a value $\alpha ^*$ of $\alpha$ for which $\xi _1(\alpha ^*)=1$ and a sequence $\alpha _n$ satisfying $\lim _{n\rightarrow \infty }\alpha _n=\alpha ^*$. It will be shown that $\lim _{n\rightarrow \infty }\xi _1(\alpha _n)=1$. Together with the information already obtained this implies that $\xi _1$ is continuous everywhere. The desired statement will be proved by contradiction. If $\xi _1(\alpha _n)$ did not converge to one then by passing to a subsequence we could assume that $\lim _{n\rightarrow \infty }\xi _1(\alpha _n)=\xi _s<1$. Consider now the sequence of solutions of the basic Selkov system with $x_n(0)=\xi _1(\alpha _n)$, $y_n(0)=1$ and $\alpha =\alpha _n$ and the solution with $x_s(0)=\xi _s$, $y_s(0)=1$ and $\alpha =\alpha ^*$. We are interested in these solutions for $t\le 0$. The sequence $(x_n,y_n)$ converges to $(x_s,y_s)$ uniformly on compact time intervals. We claim that $(x_s,y_s)$ lies on the centre manifold of $P_1$ for $\alpha =\alpha ^*$. If $(x_s,y_s)$ lies to the left of the centre manifold then it reaches negative values of *x* for finite negative values of *t*. Then for *n* sufficiently large the solutions $(x_n,y_n)$ would do the same, a contradiction. If $(x_s,y_s)$ lies to the right of the centre manifold then it must reach values of *x* greater than $\xi _s$ for finite negative values of *t*. Then for *n* sufficiently large the solutions $(x_n,y_n)$ would do the same, a contradiction. The conclusion is that the solution $(x_s,y_s)$ lies on the centre manifold and hence $\xi _1(\alpha ^*)<1$, in contradiction to the definition of $\alpha ^*$. It has thus been proved that $\xi _1$ is continuous. A similar argument shows that $\xi _2$ is continuous. Hence $\xi _1-\xi _2$ is continuous. $\square$

### Lemma 3

The function $\xi _1-\xi _2$ describing the separation of the points where the centre manifolds of $P_1$ and $P_3$ reach $y=1$ is positive for $0<\alpha \le \alpha _0$ and negative for $\alpha$ sufficiently large. There exists an $\alpha _1$ with $\xi _1(\alpha _1)= \xi _2(\alpha _1)$.

### Proof

Suppose that for a given value of $\alpha$ we have $(\xi _1-\xi _2)(\alpha )\le 0$. The region of the Poincaré compactification bounded by the parts of the centre manifolds of $P_1$ and $P_3$ ending on *L* and the part of *L* between them and above the centre manifold of $P_3$ is invariant under evolution backwards in time. Consider the solution obtained by backward time evolution of a point in this region other than the steady state. By Poincaré–Bendixson theory its $\alpha$-limit set must be a steady state or a periodic solution. If $\alpha \le \alpha _0$ this leads to a contradiction, because in that case no periodic solutions exist and the positive steady state is a sink. Thus we can conclude that the function $\xi _1-\xi _2$ is positive for $0<\alpha \le \alpha _0$.

Next we investigate the behaviour of solutions for $\alpha$ large. The following calculations were inspired by a transformation introduced in Merkin et al. (1987) in the case $\gamma =2$. It is given by $\mu =\alpha ^{-\frac{1}{\gamma }}$, ${\tilde{x}}=\alpha ^{\frac{\gamma -1}{\gamma }}x$, ${\tilde{y}}=\alpha ^{-\frac{1}{\gamma }}y$ and ${\tilde{\tau }}=\alpha \tau$. The equations become

11 $$\begin{aligned} \frac{d{\tilde{x}}}{d{\tilde{\tau }}}= & {} \mu -{\tilde{x}}{\tilde{y}}^\gamma , \end{aligned}$$

12 $$\begin{aligned} \frac{d{\tilde{y}}}{d{\tilde{\tau }}}= & {} {\tilde{x}}{\tilde{y}}^\gamma -{\tilde{y}} \end{aligned}$$

and we are interested in the limit $\mu \rightarrow 0$. A Poincaré compactification of this system was carried out in Merkin et al. (1987). After a suitable rescaling this leads to a system in the standard form of a fast-slow system in GSPT. [For background on GSPT we refer to Kuehn (2015).] Unfortunately in this system the important property of normal hyperbolicity breaks down at the point corresponding to $P_3$. It turns out that this problem can be got around by using the transformations introduced in Brechmann and Rendall (2018) to treat the behaviour of solutions for *x* large. These can be summed up by defining ${\bar{y}}=x^{-\frac{1}{\gamma }}y^{\frac{1}{\gamma }}$ and ${\bar{z}}=x^{-\frac{1}{\gamma }}y^{-\frac{\gamma -1}{\gamma }}$ and choosing a time coordinate *s* satisfying $\frac{ds}{d\tau }=\frac{1}{\gamma }xy^{\gamma -1}$. This transforms the basic Selkov system into system (12) and (13) of Brechmann and Rendall (2018). Now introduce $\epsilon =\alpha ^{-1}$ and ${\bar{w}}=\alpha ({\bar{z}}-1)$. Then, denoting the derivative with respect to *s* by a prime, we get the system

13 $$\begin{aligned} {\bar{y}}'= & {} -\gamma {\bar{y}}{\bar{w}} -{\bar{y}}\epsilon ^{-1}[(1+\epsilon {\bar{w}})^\gamma -1-\gamma \epsilon {\bar{w}}] +{\bar{y}}^{\gamma +1}\nonumber \\&-\,{\bar{y}}^\gamma (1+\epsilon {\bar{w}})^{\gamma +1}, \end{aligned}$$

14 $$\begin{aligned} \epsilon {\bar{w}}'= & {} \gamma (\gamma -1){\bar{w}} -(\gamma -1){\bar{y}}^\gamma (1+\epsilon {\bar{w}})\nonumber \\&+\,(\gamma -1)\epsilon ^{-1}[(1+\epsilon {\bar{w}})^{\gamma +1} -1-\epsilon (\gamma +1){\bar{w}}]\nonumber \\&-\,{\bar{y}}^{\gamma -1}(1+\epsilon {\bar{w}})^{\gamma +2} +\gamma {\bar{y}}^\gamma (1+\epsilon {\bar{w}}). \end{aligned}$$

Note that, due to cancellations in the expressions in square brackets, this system depends smoothly on $\epsilon$ at $\epsilon =0$ and in fact the apparently singular term even vanishes as $\epsilon \rightarrow 0$. The critical manifold has the equation $\gamma (\gamma -1){\bar{w}}={\bar{y}}^{\gamma -1}-{\bar{y}}^\gamma ={\bar{y}}^{\gamma -1}(1-{\bar{y}})$. The derivative of the right hand side of the Eq. (14) with respect to ${\bar{w}}$, evaluated at $\epsilon =0$, is $\gamma (\gamma -1)$. Thus the critical manifold is normally hyperbolic repelling. [For the terminology see Kuehn (2015).] This implies that when the system (13) and (14) is restricted to the slow manifold its limit as $\epsilon \rightarrow 0$ becomes regular.

The evolution equation on the critical manifold is

$$\begin{aligned} \frac{d{\bar{y}}}{ds}=-\frac{\gamma }{\gamma -1}{\bar{y}}^\gamma (1-{\bar{y}}). \end{aligned}$$

On the critical manifold there are two steady states, a source and a sink. They are connected by a heteroclinic orbit. For $\epsilon$ small and positive the critical manifold perturbs to a one-dimensional invariant manifold, which is the restriction of the slow manifold to that value of $\epsilon$. The points (1, 0) and (0, 0) are steady states of (13) and (14) for all values of $\epsilon$. We claim that for $\epsilon$ small they lie on the slow manifold and hence on the invariant manifold mentioned above. If we consider the extended system obtained by adjoining the equation $\epsilon '=0$ to the system (13) and (14) then the slow manifold is a centre manifold of each point of the critical manifold with respect to the extended system. Since for a steady state of a dynamical system any steady state sufficiently close to it must lie on any centre manifold of the original point the claim follows. For $(1,0,\epsilon )$ and $(0,0,\epsilon )$ are close to (1, 0, 0) and (0, 0, 0), respectively when $\epsilon$ is small. The steady state at ${\bar{y}}=1$ is hyperbolic and so perturbs to a hyperbolic source. The steady state at ${\bar{y}}=0$ continues to exist. There are no other steady states between these two because we know that there is only one positive steady state for $\epsilon >0$ due to the uniqueness of $P_0$. It follows that there is also a connection between the positive steady state $P_0$ of the Selkov system and the point $P_3$ on the boundary for $\alpha$ sufficiently large. (The direction of the flow on the connecting orbit is determined by the fact that $P_0$ is a hyperbolic source.) In other words, when $\alpha$ is sufficiently large the centre manifold of $P_3$ converges to the positive steady state in the past. This means that $\xi _2(\alpha )=1$. On the other hand, since the positive steady state is a source in this case the centre manifold of $P_1$ cannot converge to the positive steady state. We conclude that $\xi _1(\alpha )<1$ and that $\xi _1-\xi _2$ is negative. By the intermediate value theorem there exists some $\alpha _1$ with $\xi _1(\alpha _1)=\xi _2(\alpha _1)$. Note that in the end the Eqs. (11)–(12) were not needed in the proof but we judged it useful to include them so as to give an indication of how the argument was found. $\square$

It turns out that the value of $\alpha$ for which the centre manifolds of $P_1$ and $P_3$ meet is unique. This follows from a monotonicity property of the dependence of the centre manifolds on $\alpha$.

### Lemma 4

The function $\xi _1-\xi _2$ describing the separation of the points where the centre manifolds of $P_1$ and $P_3$ reach $y=1$ is strictly decreasing and has a unique zero.

### Proof

For this proof it is convenient to think of *y* as a function of *x* for a given solution. Suppose that a solution *y*(*x*) for a parameter $\alpha$ and a solution ${\hat{y}}(x)$ for a parameter ${\hat{\alpha }}<\alpha$ satisfy $y(x_1)={\hat{y}}(x_1)$ for some $x_1$. Then ${\hat{y}}'(x_1)<0$ and $|{\hat{y}}'(x_1)|<|y'(x_1)|$. Thus if ${\hat{y}}(x_2)>y(x_2)$ for some $x_2$ it follows that ${\hat{y}}(x)\ge y(x)$ for all $x\ge x_2$. Similarly, if ${\hat{y}}(x_3)<y(x_3)$ for some $x_3$ it follows that ${\hat{y}}(x)\le y(x)$ for all $x\le x_3$ The leading order approximation to the centre manifold of $P_3$ is given by ${\bar{z}}=1+\nu _1{\bar{y}}^{\gamma -1}+\ldots$ where $\nu _1=\frac{1}{\alpha \gamma (\gamma -1)}$. This translates [in terms of variables $Y={\bar{y}}^\gamma$, $Z={\bar{y}}^{\gamma -1}{\bar{z}}$ used in Brechmann and Rendall (2018)] to $Z=Y^{\frac{\gamma -1}{\gamma }}+\nu _1Y^{\frac{2(\gamma -1)}{\gamma }}\ldots$ and $x=y^{1-\gamma }-\gamma \nu _1+\ldots$. Putting these things together shows that when $\alpha$ is reduced the intersection of the centre manifold of $P_3$ with the line $y=1$ moves to the left. To obtain information about the position of the centre manifold of $P_1$ in its dependence on $\alpha$ it is necessary to determine one more order in the expansion of the centre manifold than was done in Brechmann and Rendall (2018). The result is $X=Z^{\gamma +1}-\gamma \alpha Z^{2\gamma +1}+\ldots$. In the original variables this gives $x=y^{-\gamma }-\gamma \alpha y^{-2\gamma }+\ldots$. When $\alpha$ is reduced *x* becomes larger for fixed *y*. This also means that *y* becomes larger for fixed *x* and this propagates to larger values of *x*. Thus the intersection of the centre manifold of $P_1$ with the line $y=1$ moves to the right. This implies that the function $\xi _1-\xi _2$ is strictly decreasing and cannot have more than one zero. $\square$

### Proof of Theorem 2

By Lemmas 3 and 4 there exists a unique $\alpha _1>\alpha _0$ for which the centre manifolds of $P_1$ and $P_3$ coincide. With this information the first statement of Theorem 2 follows immediately from the first statement of Theorem 1. For $\alpha _0<\alpha <\alpha _1$ the positive steady state is unstable and there is no heteroclinic cycle at infinity. It follows from the Poincaré–Bendixson theorem that the $\omega$-limit set of a solution which starts near the steady state but is not the steady state itself must be a periodic solution. In particular, a periodic solution exists and we are in the second case of Theorem 1. Thus the second statement of Theorem 2 holds. If $\alpha >\alpha _1$ then there is again no heteroclinic cycle at infinity. The $\alpha$-limit set of the solution on the centre manifold of $P_3$ must then, by the Poincaré–Bendixson theorem, be either a periodic solution or the positive steady state. Moreover, if a periodic solution exists then only the first possibility can occur. Since, however, it follows from Brechmann and Rendall (2018) that any periodic solution which exists is stable the first possibility is ruled out. There can be no periodic solution and the third case of Theorem 1 of must be realised. This completes the proof of the third statement. Finally, the fourth statement will be proved by contradiction. Let $\beta _i$ be a sequence tending to $\alpha _1$ from below. For a given *i* the system with parameter $\beta _i$ has a unique periodic solution and there is a unique point in its image of the form $(1,z_i)$ with $z_i>1$. If this sequence did not tend to infinity then it would have a convergent subsequence. Thus after passing to a subsequence $z_i$ tends to a finite limit $z^*$. The periodic solutions through the points $(1,z_i)$ converge to a solution through the point $(1,z^*)$, which is a periodic solution of the system with parameter value $\alpha _1$. This contradicts the fact that there are no such solutions. $\square$

## The Michaelis–Menten system

In the system (7) and (8) the *x*-axis is an invariant manifold of the flow and the vector field is directed towards positive values of *x* on the *y*-axis. For each fixed choice of the parameters with $\nu <1$ there is a unique positive steady state at $\left( \frac{1+\beta \nu }{1-\nu },1\right)$. For $\nu \ge 1$ there is no positive steady state. Linearizing the system about the steady state leads to the Jacobian

$$\begin{aligned} J=\left[ {\begin{array}{cc} -\frac{(1-\nu )^2}{1+\beta \nu } &{} -\gamma \left( \frac{1-\nu }{1+\beta \nu }\right) \\ \alpha \frac{(1-\nu )^2}{1+\beta \nu } &{} \alpha \left( \gamma \left( \frac{1-\nu }{1+\beta \nu }\right) -1\right) \\ \end{array}} \right] . \end{aligned}$$

The determinant of *J* is $\alpha \frac{(1-\nu )^2}{1+\beta \nu }$ which is always positive. Thus the stability of the steady state is determined by the trace of *J*, which is

$$\begin{aligned} \alpha \left[ \gamma \left( \frac{1-\nu }{1+\beta \nu }\right) -1\right] -\frac{(1-\nu )^2}{1+\beta \nu }. \end{aligned}$$

If $\gamma \le \frac{1+\beta \nu }{1-\nu }$ then the trace of *J* is negative for all values of $\alpha$ and the steady state is always stable. If $\gamma >\frac{1+\beta \nu }{1-\nu }$ define $\alpha _0=\frac{(1-\nu )^2}{\gamma (1-\nu )-(1+\beta \nu )}$. Then for $\alpha <\alpha _0$ the trace of *J* is negative and the steady state is asymptotically stable while for $\alpha >\alpha _0$ the trace of *J* is positive and the steady state is a source. For $\alpha =\alpha _0$ there is a pair of imaginary eigenvalues. If we consider the real part of the eigenvalues as a function of $\alpha$ then it passes through zero when $\alpha =\alpha _0$ and its derivative with respect to $\alpha$ at that point is non-zero. Thus a Hopf bifurcation occurs.

In the limiting case $\nu =0$ it was shown in Brechmann and Rendall (2018) that the Hopf bifurcation is supercritical so that there exists a stable periodic solution for any $\alpha$ slightly greater than $\alpha _0$. The computation of the Lyapunov number required to obtain this conclusion becomes considerably more complicated for $\nu >0$. Rather than trying to do this in general we will confine ourselves to obtaining some information for restricted sets of parameters. The Lyapunov number of the Hopf bifurcation is a function of the parameters $\alpha$, $\beta$, $\gamma$ and $\nu$ and we are interested in its sign. A general formula for this quantity is given in Sect. 4.4 of Perko (2001). It is of the form $\frac{-3\pi }{2b\Delta ^{3/2}}f$, where the first factor is positive in the present case and *f* is a function of $(\alpha ,\beta ,\gamma ,\nu )$ which is negative when $\nu =0$. This shows that in that case the Hopf bifurcation is supercritical. For $\nu$ small and positive *f* is still negative and the bifurcation supercritical. It will now be proved that there also exist parameters for which *f* is positive, so that there exists a subcritical Hopf bifurcation. In that case there exist unstable periodic solutions for $\alpha$ slightly less than $\alpha _0$. Note for comparison that it was shown in Brechmann and Rendall (2018) that for $\nu =0$ unstable periodic solutions never exist. It suffices to treat the case $\beta =0$ since an example with $\beta$ small and positive follows by continuity. Since we are only looking for some example we can also restrict to the case $\gamma =2$.

With a suitable normalization the function *f* is of the following form.

$$\begin{aligned}&\alpha (1-\nu )^2(-a_{11}^2+2\alpha a_{11}a_{02})\\&\quad +\,2(1-\nu )(\alpha ^2a_{11}^2-\alpha a_{11}(a_{02}+a_{20}))\\&\quad +\,\alpha ^2(1-\nu )^2(a_{11}a_{02}-2\alpha a_{02}^2) +2\alpha (1-\nu )^2(\alpha ^2a_{02}^2-a_{20}a_{02})\\&\quad +\,4(1-\nu )(-a_{20}^2+\alpha ^2a_{20}a_{02}) +4(2\alpha a_{20}^2-\alpha ^2a_{11}a_{20})\\&\quad +\,(2\alpha (1-\nu )+2(1-\nu )^2)(-\alpha ^2a_{11}a_{02}+a_{11}a_{20}) +(1-\nu )^2[2\alpha -(1-\nu )]\\&\quad \times \,[3(-\alpha ^2(1-\nu )a_{03}+2 a_{30})+2(1-\nu )(-a_{21}+\alpha a_{12}) +\alpha ((1-\nu )a_{12}-2a_{21})] \end{aligned}$$

Here the notation $a_{ij}$ is taken from Perko (2001). In order that there exist a bifurcation a restriction on $\nu$ must be satisfied and in the case $\gamma =2$ it is given by $\nu <\frac{1}{2}$. Consider now the limit $\nu \rightarrow \frac{1}{2}$. Since $\alpha =\frac{(1-\nu )^2}{1-2\nu }$ at the bifurcation point it tends to infinity in this limit. The highest power of $\alpha$ in the above expression is $\alpha ^3$ and two terms containing $\alpha ^3$ cancel. Substituting in the expression for the bifurcation point gives a function depending on $\nu$ alone and we want to examine its behaviour near $\nu =\frac{1}{2}$. To do this it suffices to retain only those terms in the above expression which contain a power of $\alpha$ which is at least two. At steady state and with $\beta =0$ we have

15 $$\begin{aligned} a_{11}=-2(1-2\nu )(1-\nu )^2,\ \ \ a_{03}=4\nu (1-2\nu )(1-\nu ). \end{aligned}$$

Hence the coefficients $a_{11}$ and $a_{03}$ contain a factor of $1-2\nu$. Thus to order $(1-2\nu )^{-2}$ we get the expression

$$\begin{aligned} \frac{1}{4}\alpha ^2[-4(\alpha a_{11})a_{02}-3(\alpha a_{03})+8a_{20}a_{02} +3a_{12}-4a_{21}]+\ldots \end{aligned}$$

The expression in square brackets tends to a positive value as $\nu \rightarrow \frac{1}{2}$. Thus the leading term in the expression for the Lyapunov number is positive for $\nu$ close to its limiting value. This proves that there are parameters for which the Hopf bifurcation is subcritical and thus that there exist unstable periodic solutions.

## The Poincaré compactification

In Brechmann and Rendall (2018) it was investigated using the Poincaré compactification in which ways solutions of (9) and (10) can tend to infinity for large times. Here we want to carry out corresponding calculations for (7) and (8). A useful preliminary step is to introduce a new time coordinate *T* satisfying $\frac{d\tau }{dT}=1+\nu y^\gamma (\beta +x)$. Then we get the system

$$\begin{aligned} \frac{dx}{dT}= & {} 1-xy^\gamma +\nu y^\gamma (\beta +x),\\ \frac{dy}{dT}= & {} \alpha [xy^\gamma -y-\nu y^{\gamma +1}(\beta +x)]. \end{aligned}$$

This makes the right hand side into a polynomial while leaving the phase portrait unchanged.

**Fig. 3:**
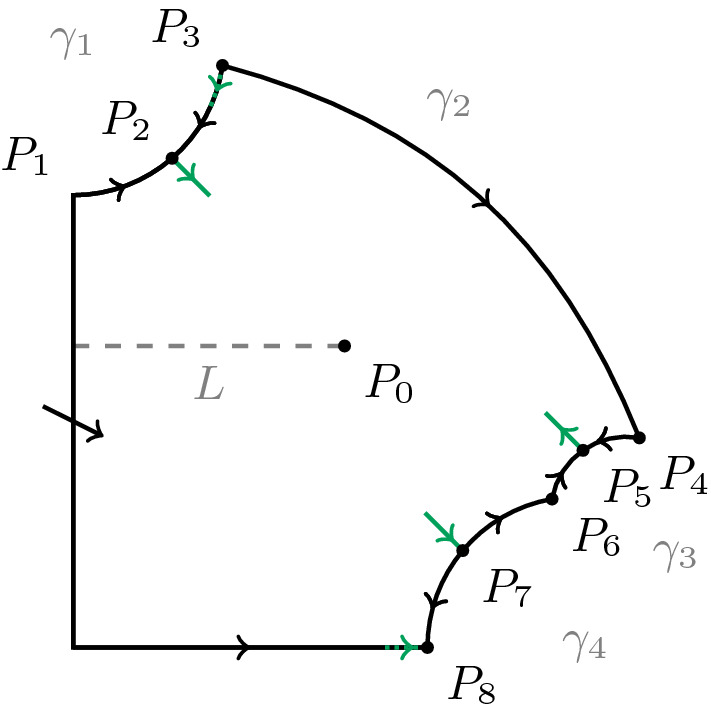
Poincaré compactification of the Michaelis–Menten system (7) and (8)

The phase portrait is more complicated than that in the case of mass action kinetics. A schematic picture of it is given in Fig. 3 and its properties are summarized in the following lemma which is the analogue of Lemma 2 in Brechmann and Rendall (2018).

### Lemma 5

Suppose that $\nu <1$. There is a smooth mapping of the closure of the positive quadrant into itself mapping the axes into themselves with the following properties. The restriction of $\phi$ to the open quadrant is a diffeomorphism onto its image. This image is a region whose closure is a compact set bounded by intervals $[0,x_0]$ and $[0,y_0]$ on the *x*- and *y*-axes and four smooth curves $\gamma _i, 1\le i\le 4$. The curve $\gamma _1$ joins the point $P_1=(0,y_0)$ with a point $P_3$ in the positive quadrant. $\gamma _2$ joins the point $P_3$ with the point $P_4$. For $3\le i\le 4$ the curve $\gamma _i$ joins the point $P_{2i-2}$ with the point $P_{2i}$ and $P_8=(x_0,0)$. The image of the dynamical system can be rescaled so as to extend smoothly to the closure of the image of $\phi$ in such a way that $P_3$ and $P_{2i}, 2\le i\le 4$, are steady states and the $\gamma _i$ and the image of the *x*-axis under $\phi$ are invariant manifolds. There are further steady states $P_2$ and $P_{2i+1}, 2\le i\le 3$, on the boundary belonging to the interior of $\gamma _1$ and $\gamma _{i+1}, 2\le i\le 3$, respectively.

The statements of Lemma 5 are proved in the remainder of this section. To analyse the case where *x* becomes large [Case 1 in the terminology of Brechmann and Rendall (2018)] introduce the variables $Y=\frac{y}{x}$, $Z=\frac{1}{x}$. Define a new time variable *t* satisfying $\frac{dt}{dT}=Z^{-\gamma -1}$. The result of the transformation is

$$\begin{aligned} \frac{dY}{dt}= & {} \alpha Y^\gamma Z+Y^{\gamma +1}Z-\alpha YZ^{\gamma +1}-YZ^{\gamma +2}\\&-\,\nu Y^{\gamma +1}(\alpha +Z)(1+\beta Z),\nonumber \\ \frac{dZ}{dt}= & {} Y^\gamma Z^2-Z^{\gamma +3}-\nu Y^\gamma Z^2(1+\beta Z). \end{aligned}$$

Both axes are invariant under the flow and the flow there is towards the origin. The linearization of the system about the origin is identically zero. Thus we do a quasihomogeneous directional blow-up. An appropriate transformation can be obtained by using a Newton polygon as in Dumortier et al. (2006). The coefficients are ${\tilde{\alpha }}=\gamma$ and ${\tilde{\beta }}=\gamma -1$. (These are the same values as occurred in the blow-up of the corresponding point for the model (9) and (10). The notation has been changed compared to that of the sources quoted by the addition of a tilde to avoid confusion with other uses of the same letters elsewhere in the present paper.) Thus we use variables ${\bar{y}}$ and ${\bar{z}}$ satisfying $(Y,Z)=({\bar{y}}^\gamma ,{\bar{y}}^{\gamma -1}{\bar{z}})$. In addition we introduce a new time coordinate *s* satisfying $\frac{ds}{dt}=\gamma ^{-1}{\bar{y}}^{\gamma ^2-1}$. The system becomes

$$\begin{aligned} \frac{d{\bar{y}}}{ds}= & {} \alpha {\bar{y}}{\bar{z}}+{\bar{y}}^{\gamma +1}{\bar{z}} -\alpha {\bar{y}}{\bar{z}}^{\gamma +1}-{\bar{y}}^\gamma {\bar{z}}^{\gamma +2}\\&-\,\nu (\alpha +{\bar{y}}^{\gamma -1}{\bar{z}}){\bar{y}}^2(1+\beta {\bar{y}}^{\gamma -1}{\bar{z}}),\\ \frac{d{\bar{z}}}{ds}= & {} -\alpha (\gamma -1){\bar{z}}^2+{\bar{y}}^{\gamma }{\bar{z}}^2 +\alpha (\gamma -1){\bar{z}}^{\gamma +2}-{\bar{y}}^{\gamma -1}{\bar{z}}^{\gamma +3}\\&+\,\nu [(\gamma -1)\alpha -{\bar{y}}^{\gamma -1}{\bar{z}}]{\bar{y}}{\bar{z}} (1+\beta {\bar{y}}^{\gamma -1}{\bar{z}}). \end{aligned}$$

Both axes are invariant under the flow. There is a steady state at the origin and one at the point (0, 1), which corresponds to $P_7$. The linearization at the origin is identically zero.

Next the centre manifold of $P_7$ will be studied. Introducing $w={\bar{z}}-1$ moves the steady state to origin. The centre subspace is given $w=\rho {\bar{y}}$ with $\rho =\frac{1-\alpha \nu }{2\alpha }$ for $\gamma =2$ and $\rho =-\frac{\nu }{\gamma }$ for $\gamma >2$.

### Lemma 6

The relation ${{\bar{y}}}'=-\frac{\gamma }{\gamma -1}{\bar{y}}^\gamma +o({\bar{y}}^\gamma )$ holds on the centre manifold of $P_7$ for all $\gamma \ge 2$.

### Proof

In the case $\gamma =2$ we have

$$\begin{aligned} {{\bar{y}}}'=\alpha {\bar{y}}{\bar{z}}-\alpha {\bar{y}}{\bar{z}}^3 -\alpha \nu {\bar{y}}^2-{\bar{y}}^2+O({\bar{y}}^3). \end{aligned}$$

Substituting the relation ${\bar{z}}=1+\rho {\bar{y}}+O({\bar{y}}^2)$ which holds on the centre manifold into this relation gives

$$\begin{aligned} {{\bar{y}}}'=\alpha \rho {\bar{y}}^2-3\alpha \rho {\bar{y}}^2 -\alpha \nu {\bar{y}}^2-{\bar{y}}^2 +O({\bar{y}}^3)=-2{\bar{y}}^2+O({\bar{y}}^3) \end{aligned}$$

and this completes the proof for $\gamma =2$. For $\gamma >2$ we use the relation

$$\begin{aligned} {{\bar{z}}}'=-{\bar{y}}^{\gamma -1} +(\gamma -1)[-\alpha {\bar{z}}^2+\alpha {\bar{z}}^{\gamma +2} +\alpha \nu {\bar{y}}{\bar{z}}]+O({\bar{y}}^\gamma ). \end{aligned}$$

Substituting this into the evolution equation for ${\bar{y}}$ gives

$$\begin{aligned} {{\bar{y}}}'=-\frac{\gamma }{\gamma -1}{\bar{y}}^{\gamma } -\frac{1}{\gamma -1}{\bar{y}}{\bar{z}}^{-1}{{\bar{z}}}'+O({\bar{y}}^{\gamma +1}). \end{aligned}$$

Suppose that we know that ${{\bar{y}}}'=O({\bar{y}}^k)$ for some *k* with $2\le k\le \gamma -1$. Then it follows that ${{\bar{z}}}'=O({\bar{y}}^{k+1})$. Hence ${{\bar{y}}}'=O({\bar{y}}^{k+1})$. After finitely many steps we get the conclusion of the lemma for $\gamma >2$. $\square$

We see that the flow on the centre manifold is towards $P_7$ and since the non-zero eigenvalue of the linearization at that point is positive $P_7$ is a topological saddle. In fact the flow on the boundary is everywhere away from $P_7$. We next blow up the origin in the coordinates $({\bar{y}},{\bar{z}})$. This time the procedure described in Dumortier et al. (2006) leads to the choice of coefficients ${\tilde{\alpha }}={\tilde{\beta }}=1$. Blow-ups in the two coordinate directions are required. The only terms in the resulting equations which will be written explicitly are those which have a direct influence on the analysis which follows. In the first transformed system, with ${\bar{y}}={\tilde{y}}_1$ and ${\bar{z}}={\tilde{y}}_1{\tilde{z}}_1$, the equations are

$$\begin{aligned} {{\tilde{y}}_1}'= & {} {\tilde{y}}_1[-\alpha (\nu -{\tilde{z}}_1){\tilde{y}}_1+\cdots ],\\ {{\tilde{z}}_1}'= & {} {\tilde{y}}_1[\gamma \alpha (\nu -{\tilde{z}}_1){\tilde{z}}_1+\cdots ]. \end{aligned}$$

A change of time coordinate eliminates the common factor ${\tilde{y}}_1$. On the boundary there is a steady state at the point $(0,\nu )$, which corresponds to $P_5$. The origin of this coordinate system corresponds to $P_4$. The terms which have been retained suffice to determine the steady states on the boundary and the linearization of the system at those points. The point $P_5$ also appears in the second transformed system but since it can be analysed in the chart corresponding to the first transformed system the second transformed system, with ${\bar{y}}={\tilde{y}}_2{\tilde{z}}_2$ and ${\bar{z}}={\tilde{z}}_2$, is only needed to analyse the steady state $P_6$ at the origin of that coordinate system. For this purpose the only terms which need to be retained are

$$\begin{aligned} {{\tilde{y}}_2}'= & {} {\tilde{z}}_2[\gamma \alpha {\tilde{y}}_2+\cdots ],\\ {{\tilde{z}}_2}'= & {} {\tilde{z}}_2[-\alpha (\gamma -1){\tilde{z}}_2+\cdots ]. \end{aligned}$$

The common factor ${\tilde{z}}_2$ can be eliminated by a change of time coordinate. The origin of this coordinate system corresponds to $P_6$. We see that in both cases, after a suitable change of time coordinate, the origin is a hyperbolic saddle.

Next the centre manifold of $P_5$ will be studied in the case $\gamma =2$. We do not expect that the case $\gamma >2$ is essentially different but since the algebra becomes significantly more complicated only the case $\gamma =2$ has been worked out. The centre subspace is parallel to the ${\tilde{y}}_1$-axis. We have ${{\tilde{z}}_1}'=O({\tilde{y}}_1^3)$ on the centre manifold and this implies that if ${\tilde{z}}_1=\nu +w$ then

$$\begin{aligned} 2\alpha \nu w=[\nu ^2(1-\nu )+2\alpha \nu ^4+2\alpha \beta \nu ^3]{\tilde{y}}_1^2+\ldots \end{aligned}$$

It follows that provided $\nu <1$ the flow on the centre manifold of $P_5$ is away from $P_5$. For the rest of the discussion we return to the case of general $\gamma$.

In the case where *x* gets large it remains to do one further quasihomogeneous directional blow-up of the origin in the (*Y*, *Z*) coordinate system. In this case $(Y,Z)=({\bar{y}}{\bar{z}}^{\gamma },{\bar{z}}^{\gamma -1})$. The time coordinate is transformed using the relation $\frac{ds}{dt}=\frac{1}{\gamma -1} {\bar{z}}^{\gamma ^2-1}$. The resulting system is

16 $$\begin{aligned} {\bar{y}}'= & {} (\gamma -1)[\alpha {\bar{y}}^\gamma -\alpha {\bar{y}} -\alpha \nu {\bar{y}}^{\gamma +1}{\bar{z}}(1+\beta {\bar{z}}^{\gamma -1})\nonumber \\&+\,{\bar{y}}^{\gamma +1}{\bar{z}}^\gamma -{\bar{y}}{\bar{z}}^{\gamma -1} -\nu {\bar{y}}^{\gamma +1}{\bar{z}}^\gamma (1+\beta {\bar{z}}^{\gamma -1})]\nonumber \\&-\,\gamma [{\bar{y}}^{\gamma +1}{\bar{z}}^\gamma -{\bar{y}}{\bar{z}}^{\gamma -1}-\nu {\bar{y}}^{\gamma +1}{\bar{z}}^\gamma (1+\beta {\bar{z}}^{\gamma -1})], \end{aligned}$$

17 $$\begin{aligned} {\bar{z}}'= & {} {\bar{y}}^\gamma {\bar{z}}^{\gamma +1} -{\bar{z}}^\gamma -\nu {\bar{y}}^\gamma {\bar{z}}^{\gamma +1} (1+\beta {\bar{z}}^{\gamma -1}). \end{aligned}$$

There is a steady state at the point (1, 0) but since it is just another representation of $P_7$ it does not need to be analysed further. The origin of this coordinate system corresponds to $P_8$. The ${\bar{z}}$-axis is a centre manifold for $P_8$ and the flow there is towards $P_8$. Since the non-zero eigenvalue of the linearization at $P_8$ is negative it can be concluded that $P_8$ is a sink.

Having completed the analysis of the case where *x* gets large we now turn to the case where where *y* gets large [Case 2 in the terminology of Brechmann and Rendall (2018)], with new variables $X=\frac{x}{y}$ and $Z=\frac{1}{y}$. The result is

$$\begin{aligned} \frac{dX}{dT}= & {} \frac{1}{Z^{\gamma +1}}[Z^{\gamma +2}-XZ+\nu Z(X+\beta Z)\\&-\,\alpha X^2Z+\alpha XZ^{\gamma +1}+\alpha \nu X (X+\beta Z)],\\ \frac{dZ}{dT}= & {} \frac{1}{Z^{\gamma +1}} [-\alpha XZ^2+\alpha Z^{\gamma +2}+\alpha \nu Z(X+\beta Z)]. \end{aligned}$$

The common factor $\frac{1}{Z^{\gamma +1}}$ can be removed by a suitable change of time coordinate satisfying $\frac{dt}{dT}=Z^{-\gamma -1}$. The linearization of the resulting system about the origin is identically zero so that it is again necessary to do a blow-up. In this case a calculation using a Newton polygon gives the exponents ${\tilde{\alpha }}=1$ and ${\tilde{\beta }}=1$. The transformation in the *X* direction uses the relation $(X,Z)=({\bar{x}}_1,{\bar{x}}_1{\bar{z}}_1)$. The resulting system is

$$\begin{aligned} \frac{d{\bar{x}}_1}{dt}= & {} {\bar{x}}_1[{\bar{x}}_1^{\gamma +1}{\bar{z}}_1^{\gamma +2} -{\bar{x}}_1{\bar{z}}_1 +\nu {\bar{x}}_1{\bar{z}}_1 (1+\beta {\bar{z}}_1)\\&-\,\alpha {\bar{x}}_1^2{\bar{z}}_1+\alpha {\bar{x}}_1^{\gamma +1}{\bar{z}}_1^{\gamma +1} +\alpha \nu {\bar{x}}_1 (1+\beta {\bar{z}}_1)],\\ \frac{d{\bar{z}}_1}{dt}= & {} {\bar{x}}_1[-{\bar{x}}_1^{\gamma }{\bar{z}}_1^{\gamma +3}+{\bar{z}}_1^2 -\nu {\bar{z}}_1^2 (1+\beta {\bar{z}}_1)]. \end{aligned}$$

The origin of this coordinate system corresponds to $P_3$. By a change of time coordinate we can remove the factor ${\bar{x}}_1$. The linearization of the system which results at the origin has one positive eigenvalue and the ${\bar{z}}_1$-axis is invariant and defines a centre manifold at that point. It can be concluded that $P_3$ is a source. If $\nu <1$ there is a steady state at the point $\left( 0,\frac{1-\nu }{\beta \nu }\right)$ which corresponds to the point $P_2$. That point is a hyperbolic saddle whose stable manifold is the ${\bar{z}}_1$-axis.

The transformation in the *Z* direction uses the relation $(X,Z)=({\bar{x}}_2{\bar{z}}_2,{\bar{z}}_2)$. The resulting system is

$$\begin{aligned} \frac{d{\bar{x}}_2}{dt}= & {} {\bar{z}}_2[{\bar{z}}_2^{\gamma }-{\bar{x}}_2 +\nu (\beta +{\bar{x}}_2)],\\ \frac{d {\bar{z}}_2}{dt}= & {} -\alpha XZ^2+\alpha Z^{\gamma +2}+\alpha \nu Z(X+\beta Z)\\= & {} {\bar{z}}_2[-\alpha {\bar{x}}_2{\bar{z}}_2^2+\alpha {\bar{z}}_2^{\gamma +1} +\alpha \nu {\bar{z}}_2(\beta +{\bar{x}}_2)]. \end{aligned}$$

The origin of this coordinate system is $P_1$. By a change of time coordinate we can remove the factor ${\bar{z}}_2$. In the system which results there is inflow on the ${\bar{z}}_2$-axis while the ${\bar{x}}_2$-axis is invariant and corresponds to the ${\bar{z}}_1$-axis in the previous system. Note that the point $P_1$ is not a steady state.

The facts which have now been collected imply strong restrictions on the possible $\omega$-limit sets of solutions. The only points of the boundary which they can contain are those on the part connecting $P_5$ and $P_7$. Poincaré–Bendixson theory implies that the $\omega$-limit set of a positive solution must be either a point (which can only be the positive steady state, $P_7$ or $P_8$), a periodic solution or a heteroclinic cycle joining $P_5$ and $P_7$. The last of these can only occur if the centre manifolds of $P_5$ and $P_7$ coincide. Note that any periodic solution or heteroclinic cycle must contain the positive steady state in its interior.

We have the following analogue of Theorem 1 of Brechmann and Rendall (2018).

### Theorem 3

There exists a positive number $\epsilon >0$ such that any solution of the Michaelis–Menten system (7) and (8) with initial data $x(0)=x_0$ and $y(0)=y_0$ which satisfies $x_0>\epsilon ^{-1}$ and $x_0y_0^\gamma <\epsilon$ has the late-time asymptotics

$$\begin{aligned} x(\tau )= & {} \tau (1+o(1)),\\ y(\tau )= & {} y_1e^{-\alpha \tau }(1+o(1)). \end{aligned}$$

for a constant $y_1$. There exists a solution, unique up to time translation, which has the asymptotic behaviour

$$\begin{aligned} x(\tau )= & {} \tau (1+o(1)),\\ y(\tau )= & {} \tau ^{-\frac{1}{\gamma -1}}(1+o(1)). \end{aligned}$$

### Proof

The proof of this theorem is very similar to that of Theorem 1 of Brechmann and Rendall (2018), whose basic structure we now recall. Any solution which starts close enough to the point $P_8$ converges to that point as $t\rightarrow \infty$. Using this information in the system (16) and (17) allows these equations for ${\bar{y}}(s)$ and ${\bar{z}}(s)$ to be integrated to leading order in the limit $s\rightarrow \infty$. The resulting asymptotic expressions can then be transformed back to the original variables $x(\tau )$ and $y(\tau )$. The only extra element is that, while in the original proof only one change of time coordinate was required, in the present case we must first transform from *s* to *T* and then from *T* to $\tau$. Since for this type of solution the time coordinates $\tau$ and *T* are asymptotically equal this extra element does not change the final answer. In particular, the parameter $\nu$ does not contribute to the leading order asymptotics. This gives the first statement of the theorem. The solution mentioned in the second statement of the theorem is a solution on the centre manifold of $P_7$. Integrate the equation for ${\bar{y}}$ in Lemma 6 in leading order in the limit $s\rightarrow \infty$ and substitute the result into the equation for ${\bar{z}}$. This provides asymptotic expressions for ${\bar{y}}(s)$ and ${\bar{z}}(s)$. Transforming these back to the variables $x(\tau )$ and $y(\tau )$ gives the second part of the theorem. $\square$

## The global phase portrait

To understand the global phase portrait it is helpful to understand the geometry of the nullclines $N_1$ and $N_2$ given by $\dot{x}=0$ and $\dot{y}=0$, respectively. We restrict consideration to the case $\nu <1$ where $N_1$ and $N_2$ intersect in a single point. The equation for $N_1$ can be expressed in the equivalent forms

$$\begin{aligned} y= & {} \left[ \frac{1}{-\beta \nu +(1-\nu )x}\right] ^{\frac{1}{\gamma }},\ \ \ x=\frac{1}{1-\nu }(y^{-\gamma }+\beta \nu ). \end{aligned}$$

Thus on $N_1$ the coordinate *y* can be expressed as a smooth function of *x* with a smooth inverse. Note, however, that while the second function is defined for all positive *y* the first is only defined for $x>\frac{\beta \nu }{1-\nu }$. The equation for $N_2$ can be expressed in the form

$$\begin{aligned} x=\frac{y^{1-\gamma }+\beta \nu y}{1-\nu y}. \end{aligned}$$

**Fig. 4:**
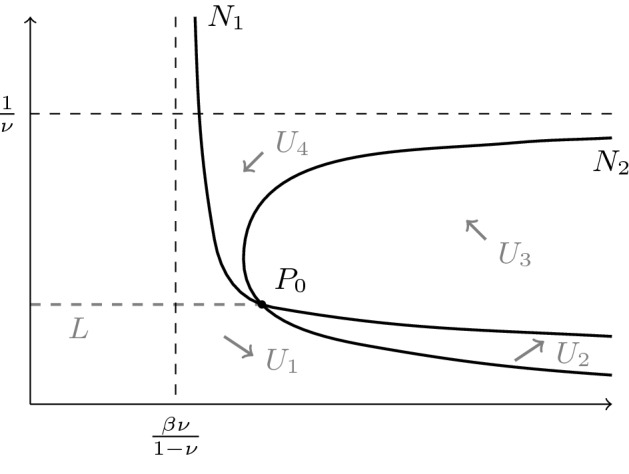
Nullclines of the Michaelis–Menten system (7) and (8)

Thus on $N_2$ the coordinate *x* can be expressed as a (locally defined) smooth function of *y*. Since *x* can be written as a function of *y* in both cases and there is only one point of intersection it is clear that the complement of $N_1\cup N_2$ is a union of the four connected components defined by the signs of $\dot{x}$ and $\dot{y}$. A schematic picture of the null clines is given in Fig. 4. As in Fig. 2 we denote the regions with the sign combinations $(+,-)$, $(+,+)$, $(-,+)$ and $(-,-)$ by $U_1$, $U_2$, $U_3$ and $U_4$, respectively. Where $\dot{y}=0$ and $\dot{x}\ne 0$ we can use the fact that $N_2$ is a graph over the *y*-axis to conclude that a solution can only pass from $U_3$ to $U_4$ and $U_1$ to $U_2$ and not the other way round. Similarly the fact that $N_1$ is a graph over the *y*-axis implies that a solution can only pass from $U_4$ to $U_1$ and $U_2$ to $U_3$ and not the other way round. Thus the possible passages between the regions $U_i$ are just as in the case with mass action kinetics. Let *L* be the part of the horizontal line segment joining the positive steady state to the *y*-axis with the endpoint on the axis excluded. The part of *L* excluding the steady state is contained in $U_1$.

### Lemma 7

In the Michaelis–Menten system each of the centre manifolds of $P_5$ and $P_7$ contains a point of *L* in its closure.

### Proof

A point on the centre manifold of $P_5$ which is sufficiently close to $P_5$ lies in the region $U_3$. If we follow a solution which lies on this manifold forwards in time then it must either tend to the positive steady state as $t\rightarrow \infty$ or it must enter $U_4$ after a finite time and in the latter case it must enter $U_1$. Once it has done so it must either tend to the positive steady state as $t\rightarrow \infty$ or it must meet *L* after a finite time. Similarly a solution on the centre manifold of $P_7$ which starts close to $P_7$ must, when followed backwards in time, either converge to the positive steady state as $t\rightarrow -\infty$ or meet *L* after a finite time. $\square$

Denote the *x*-coordinates of the points of *L* in the closure of the centre manifolds of $P_5$ and $P_7$ for given value of $\alpha$ and $\nu$ by $\xi _1(\alpha ,\nu )$ and $\xi _2(\alpha ,\nu )$.

### Lemma 8

The function $\xi _1-\xi _2$ describing the separation of the points where the centre manifolds of $P_5$ and $P_7$ reach $y=1$ is continuous.

### Proof

The proof is similar to that of Lemma 2. The essential facts which must be used are that any solution which approaches $P_5$ close enough in the past time direction and does not lie on the centre manifold of $P_5$ cannot remain close to $P_5$ forever and the corresponding statement with ’past’ replaced by ’future’ and $P_5$ by $P_7$. $\square$

### Lemma 9

There are pairs of parameters $(\alpha ,\nu )$ for which the function $\xi _1-\xi _2$ describing the separation of the points where the centre manifolds of $P_5$ and $P_7$ reach $y=1$ is negative. For $\nu >0$ fixed and $0<\alpha \le \alpha _0$ if there exists no unstable periodic solution then $\xi _1-\xi _2$ is positive. If there is some $\alpha \le \alpha _0$ for which no periodic solutions exist then there exists an $\alpha _1$ with $\xi _1(\alpha _1,\nu )= \xi _2(\alpha _1,\nu )$.

### Proof

For $\alpha$ sufficiently large the positive steady state is a source and thus $\xi _1(\alpha ,\nu )<1$. Thus in order to prove the first part of the lemma it suffices to show that for some $(\alpha ,\nu )$ we have $\xi _2(\alpha ,\nu )=1$. To prove this we proceed as in the case of mass action kinetics. First the system is transformed to the coordinates $({\bar{y}},{\bar{z}})$ and then the quantities $\epsilon$ and ${\bar{w}}$ are introduced. The right hand side of each equation in the Michaelis–Menten case is the sum of the right hand side of the corresponding equation in the mass action case and an expression which can be written as $\epsilon ^{-1}\nu$ times a function which is regular in the limit $\epsilon \rightarrow 0$. Fixing $\nu$ and letting $\epsilon$ tend to zero would cause this term to explode. Instead we let $\epsilon$ and $\nu$ tend to zero in such a way that $\nu =\epsilon ^2$. Then the second summand behaves in a smooth manner as $\epsilon \rightarrow 0$ and in fact tends to zero. Thus proceeding in the same way as in the proof of Lemma 3 gives the first conclusion. For the second part we can again proceed as in the proof of Lemma 3. The difference is that while in the mass action case we knew that there was no unstable periodic solution in the Michaelis–Menten case we have to assume it. $\square$

Note that for the choice of parameters in the first part of Lemma 9 there is a heteroclinic orbit joining the positive steady state to the point $P_7$. It follows that for these values of the parameters no periodic solutions exist. This is because a periodic solution would have to contain the positive steady state in its interior and therefore would have to cross the heteroclinic orbit.

There is no straightforward generalization of the monotonicity result of Lemma 4 to the Michaelis–Menten case. The proof of monotonicity fails for the centre manifold of $P_5$ since it may pass through the region $U_4$. For this reason even in a case where the existence of a zero of $\xi _1-\xi _2$ can be proved we do not get its uniqueness, Moreover, we do not get the analogue of the stability statement in the mass action case. It is possible to do a calculation analogous to that done to determine the stability of the heteroclinic cycle in Brechmann and Rendall (2018). Unfortunately in the estimate for the return map the power $\gamma$ is replaced by the power one and this gives no information about stability. The following theorem sums up the results obtained.

### Theorem 4

The Michaelis–Menten system (7) and (8) has the following properties.

1. For each choice of the parameters $(\alpha ,\nu )$ with $\nu <1$ the unique positive steady state has coordinates $\left( \frac{1+\beta \nu }{1-\nu },1\right)$.
2. If $\gamma \le \frac{1+\beta \nu }{1-\nu }$ the steady state is stable. Otherwise if $\alpha <\alpha _0=\frac{(1-\nu )^2}{\gamma (1-\nu )-(1+\beta \nu )}$ it is stable and for $\alpha >\alpha _0$ unstable.
3. For $\alpha =\alpha _0$ a Hopf bifurcation occurs. Parameters can be chosen so as to make it supercritical or subcritical.
4. For given $(\alpha ,\nu )$ there exist positive numbers $x_0$ and $y_0$ such that if a solution satisfies $x(t)\ge x_0$ and $y(t)\le y_0$ at some time *t* then it has the late time asymptotics described in Theorem 3.
5. For $\gamma =2$ there exists a choice of positive parameters $\alpha$ and $\nu$ for which all solutions other than the steady state have the late time asymptotics described in Theorem 3.
6. If for $\gamma =2$ and given $\nu$ there exist no periodic solutions for $\alpha$ sufficiently small then there exists a heteroclinic cycle passing through the steady states $P_7$ and $P_5$ in that order.

It should be noted that it has not been shown here whether the case described in point 6. ever occurs.

## Conclusions and outlook

It has been shown that the basic Selkov system admits solutions with unbounded oscillations and that the diameter of the image of a periodic solution can tend to infinity as $\alpha$ approaches a finite limit, thus completing the results of Brechmann and Rendall (2018) on that system and rigorously confirming a claim made in Selkov (1968). Note that some statements related to this issue have been made in Erneux (2018) but that reference does not contain rigorous proofs of those statements. One remaining question is that of the rate with which the diameter of the image of the periodic solution tends to infinity as the critical parameter value $\alpha _1$ is approached. A suggestion for this has been made in Merkin et al. (1987) for the case $\gamma =2$ but there is neither a rigorous proof that this suggestion is correct nor a generalization of the statement to higher values of $\gamma$.

It was also investigated which properties of the basic Selkov system persist in the Michaelis–Menten system from which Selkov derived his basic model. Partial results were obtained and it was shown in particular that the Michaelis–Menten system has unbounded solutions which are eventually monotone for all parameter values. The question of whether the five-dimensional system from which the Michaelis–Menten system itself was derived has unbounded solutions remains open. It was shown that for suitable parameter values unstable periodic solutions of the Michaelis–Menten system exist. It was left open whether there exist unbounded oscillatory solutions or periodic solutions whose images have arbitrarily large diameter for bounded ranges of the parameters.

The unbounded solutions cast doubt on the suitability of the Selkov model for describing glycolytic oscillations. An alternative model often preferred to the Selkov model is that of Goldbeter and Lefever (1972). There the amplitude of the periodic solutions created in a Hopf bifurcation increases to a maximum before decreasing again to zero at a point where the periodic solutions vanish again in a second Hopf bifurcation. It has been proved by d’Onofrio (2011), on the basis of an analysis of a more general class of systems in Othmer and Aldridge (1978), that all solutions of the Goldbeter-Lefever model are bounded. Further aspects of the dynamics of solutions of that model have been studied in d’Onofrio (2011) and a sophisticated analysis of some of its properties has been carried out in Kosiuk and Szmolyan (2011).

The questions of the origin of the unbounded solutions and how they could be eliminated by modifying the system have been discussed in Merkin et al. (1986). The origin of the unbounded growth can be seen in the constant source term in the equation for *x*. This corresponds to an unlimited supply of the substrate. In Merkin et al. (1986) this is called the pooled chemical approximation. If this is replaced by a mechanism where the substrate is formed from a precursor which itself is limited in quantity then the oscillations only grow within a finite time period before decaying again. An alternative modification is to introduce an additional uncatalysed conversion of the substrate into the product. The resulting system is called the (cubic) autocatalator. According to the analysis of Merkin et al. (1986) this leads to a situation similar to that described above for the Goldbeter-Lefever model and the unbounded oscillations are absent. Some aspects of this type of model have been analysed rigorously in Gucwa and Szmolyan (2009).

The Selkov system and other related ones are model cases for understanding oscillations in biological and chemical systems. The study of equations of this type raises a number of issues. In what ways can heuristic and numerical results be made into rigorous theorems? How can we understand the relations between the choices made in modelling and the relevance of the resulting models for the applications? The present paper is intended as a contribution to the clarification of these issues.
